# A new GABAergic somatostatin projection from the BNST onto accumbal parvalbumin neurons controls anxiety

**DOI:** 10.1038/s41380-020-0816-3

**Published:** 2020-06-18

**Authors:** Qian Xiao, Xinyi Zhou, Pengfei Wei, Li Xie, Yaning Han, Jie Wang, Aoling Cai, Fuqiang Xu, Jie Tu, Liping Wang

**Affiliations:** 1 Shenzhen Key Lab of Neuropsychiatric Modulation, Guangdong Provincial Key Laboratory of Brain Connectome and Behavior, CAS Key Laboratory of Brain Connectome and Manipulation, the Brain Cognition and Brain Disease Institute (BCBDI), Shenzhen Institutes of Advanced Technology, Chinese Academy of Sciences (CAS); Shenzhen-Hong Kong Institute of Brain Science-Shenzhen Fundamental Research Institutions, Shenzhen, 518055 China; 2 University of Chinese Academy of Sciences, Beijing, 100049 PR China; 3 Center of Brain Science, State Key Laboratory of Magnetic Resonance and Atomic and Molecular Physics, National Center for Magnetic Resonance in Wuhan, Key Laboratory of Magnetic Resonance in Biological Systems, Wuhan Institute of Physics and Mathematics, Innovation Academy for Precision Measurement Science and Technology, Chinese Academy of Sciences (CAS), Wuhan, 430071 PR China; 4 Wuhan National Laboratory for Optoelectronics, Huazhong University of Science and Technology, Wuhan, 430074 PR China

**Keywords:** Neuroscience, Physiology

## Abstract

The prevailing view is that parvalbumin (PV) interneurons play modulatory roles in emotional response through local medium spiny projection neurons (MSNs). Here, we show that PV activity within the nucleus accumbens shell (sNAc) is required for producing anxiety-like avoidance when mice are under anxiogenic situations. Firing rates of sNAc^PV^ neurons were negatively correlated to exploration time in open arms (threatening environment). In addition, sNAc^PV^ neurons exhibited high excitability in a chronic stress mouse model, which generated excessive maladaptive avoidance behavior in an anxiogenic context. We also discovered a novel GABAergic pathway from the anterior dorsal bed nuclei of stria terminalis (adBNST) to sNAc^PV^ neurons. Optogenetic activation of these afferent terminals in sNAc produced an anxiolytic effect via GABA transmission. Next, we further demonstrated that chronic stressors attenuated the inhibitory synaptic transmission at adBNST^GABA^ → sNAc^PV^ synapses, which in turn explains the hyperexcitability of sNAc PV neurons on stressed models. Therefore, activation of these GABAergic afferents in sNAc rescued the excessive avoidance behavior related to an anxious state. Finally, we identified that the majority GABAergic input neurons, which innervate sNAc^PV^ cells, were expressing somatostatin (SOM), and also revealed that coordination between SOM- and PV- cells functioning in the BNST → NAc circuit has an inhibitory influence on anxiety-like responses. Our findings provide a potentially neurobiological basis for therapeutic interventions in pathological anxiety.

## Introduction

Stressors and stress responses are critical for guiding both approach and avoidance behaviors in animals and humans. Exposure to chronic, unpredictable stressors leads to increased anxiety responses, including excessive avoidance behavior, and this exposure has been adopted to study anxious state-related behaviors [1, 2]. The bed nucleus of the stria terminalis (BNST), a subregion of the extended amygdala, is a critical node in the stress response [3, 4]. Recent work on human drug addiction has also demonstrated a role of BNST in withdrawal-related anxiety and relapse [4], indicating an intrinsic link between this stress response region and the reward system. The nucleus accumbens (NAc) is a vital component in the reward circuitry [5–7], which responds to stress signals [8, 9] and has a dominant effect on anxiety regulation [10]. Based on the findings that both BNST and NAc are engaged in stress, anxiety, and addiction, it is reasonable to think that there may be interactions between these two regions. However, with the exception of one 15-year-old anatomical observation [11], any functional connectivity between these two areas, as well as information about the neurochemical composition of the BNST-NAc projections, remains unexplored. Furthermore, GABAergic efferents originating from the BNST are predominantly sent downstream [12, 13], however, the nature and function of any GABAergic input to NAc is unknown.

Medium spiny neurons that express dopamine 1 and 2 receptors (D1- and D2-MSNs) are the predominant neural population in the NAc. Regarding anxiety regulation, it is accepted that D1-MSNs are not involved in anxiety-like behavior, but play roles in modulating reward-related responses [14, 15] whereas D2-MSNs may regulate anxiety-like aversion or avoidance behavior [16]. However, the role of D2-MSNs are not entirely clear because other work points to a role in reward seeking, but not anxiety-like behavior [17, 18]. Based on these very different findings, we predicted that there is another neuronal type within the NAc that contributes to anxiety-like behavior. One possibility is PV GABAergic interneurons, which comprise only 3-4% of all neurons in the NAc [19–21]. In other brain regions, these neuron regulate fear response [22], anxiolysis [21], alcohol addiction [23], and reward seeking [24]. However, we know less about the function of accumbal PV neurons and the inputs they receive, and nothing about any possible role in anxiety-related behavior.

We addressed these important questions regarding the neural mechanisms underlying the expression of avoidance of anxiogenic stimuli in both healthy and pathological anxiety models. Combining functional MRI signaling, GCaMP-based fiber photometry recording, genetically modified virus tracing and both optogenetic and chemogenetic neuronal manipulations, we show that in an anxious state, functional connectivity between BNST and NAc was increased. PV neurons within the NAc shell (sNAc) exhibited high excitability in a chronic stress mouse model that displayed excessive maladaptive avoidance during anxiogenic stimuli, and further, activation of these accumbal PV neurons promoted an avoidance coping response in healthy mice. A new GABAergic somatostatin (SOM) afferent from the anterior dorsal BNST (adBNST) was uncovered, which directly innervates sNAc^PV^ neurons. Optogenetic activation of these GABAergic terminals in sNAc produced an anxiolytic effect, which was mediated by sNAc^PV^ cells. Inhibitory projections from adBNST attenuated sNAc ^PV^ neurons on the chronically stressed mice, and thus, activation of these inhibitory inputs from adBNST to sNAc rescued the excessively anxious state of the stressed mice.

Therefore, our results reveal a previously undescribed circuit mechanism, defined by neuronal type, that shapes the coordination between BNST^SOM^ and NAc^PV^ cells in response to anxiogenic stimuli under both physiological and pathological conditions.

## Materials and methods

### Mouse models

All experiments were approved by the Shenzhen Institutes of Advanced Technology, Chinese Academy of Sciences Research Committee, and all experimental procedures involving animals were carried out in strict accordance with the Research Committee’s animal use guidelines. Surgery was performed under full anesthesia, and every effort was made to minimize animal suffering. Male and female mice (6–14 weeks) were used in this study. We used the following mouse lines: PV-Cre (B6;129P2-Pvalbtm1(cre)Arbr/J, Jackson Laboratory, stock No.008069), D1-Cre (B6.Cg-Tg(Drd1a-cre)262Gsat/Mmcd, MMRRC, 030989-UCD), D2-Cre (B6.FVB(Cg)-Tg(Drd2-cre)ER44Gsat/Mmcd, MMRRC,032108-UCD), and SOM-Cre (STOCK Ssttm 2.1(cre) Zjh/J, Jackson Laboratory, stock No.013044). C57BL/6J wild-type mice were also used.

### Behavioral tests

For all animal experiments, littermate mice were randomly assigned to experimental group and were identified by unique identification number. Experimenters were blind to experimental group allocation. In addition, data analysts were also blind to experimental conditions. Groups of mice were age-matched (8–14 weeks). All mice were handled for 15–30 min per day for 3 days before behavioral assays to reduce stress introduced by contact with experimenter.

#### Elevated plus maze test

A plastic elevated plus-maze consisting of a central platform (5 × 5 cm) with two white open arms (25 × 5 × 25 cm) and two white closed arms (25 × 5 × 25 cm) extending from it in a plus shape was used. The maze was elevated 65 cm above the floor. Mice were individually placed in the center with their heads facing a closed arm. The number of entries and the amount of time spent in each arm type were recorded.

#### Open-field test

A plastic open field chamber (50 × 50 cm) was used and conceptually divided into a central field (25 × 25 cm) and a peripheral field for analysis. Each mouse was placed in the peripheral field at the start of each test. The number of entries and the amount of time spent in center were recorded. Mice locations were monitored/tracked using Anymaze software.

#### Unpredictable chronic mild stress procedure (UCMS)

The UCMS protocol was performed as previously described [25, 26] with modification. Mice were exposed to environmental stressors for three weeks. One of the following stressors were presented during each daily session in a random order over three weeks: (i) restraint, where each mouse was placed in a tube (50 mL) for 2 h without access to food or water, (ii) a wet environment where water was added (such that bedding was damp but not overly wet) to a housing cage containing mice for 12 h sessions, and (iii) squeezing, where four mice were housed into a box (3 × 5 × 7 cm) for 2 h, without access to food or water.

#### Novel object interaction

Mice were habituated for 5 min to an empty box (40 × 40 × 30 cm) with white floor and walls. A novel object (a Rubik’s cube) was placed in the center of the box 24 h later and mouse interactions with the object were recorded in one 5-min test session. During optogenetic stimulation experiments, light was delivered during the test sessions.

#### Three-chamber social interaction tests

A three-chambered apparatus (60 × 40 × 25 cm) with a central chamber (20 cm wide) and two side chambers (each 20 cm wide) was used. An empty housing cage was put inside each side chamber and the test mouse was placed in the central chamber and allowed to freely explore the test arena for a 5 min habituation period. Then, in a second phase (sociability), the mouse was gently guided back to the central chamber, the two side-chamber entrances were blocked and then a ‘stranger’ mouse (Stranger 1) was placed in one side chamber. Both entrances were then opened to allow the test mouse to explore the new environment freely for five min. In a third phase (social novelty), a second stranger (Stranger 2) was placed in the other empty side chamber and the test mouse again could freely explore all three chambers for five min. All stranger mice were males of the same age. For optogenetic stimulation experiments, light was delivered during the second and third phases.

### Fiber photometry

Fiber photometry allows for real-time excitation and recording of fluorescence from genetic encoded calcium indicators in freely moving mice. Mice were habituated to the fiber patch cord for at least 15 min per day for 3 days before tests were conducted inside home cages. The fiber photometry system (ThinkerTech, Nanjing, China) consisted of an excitation LED light (480 nm; CREE XPE), reflected off a dichroic mirror with a 435–488 nm reflection band and a 502–730 nm transmission band (Edmund, Inc), coupled into a 200 μm 0.37 NA optical fiber (Thorlabs, Inc) by an objective lens. The laser intensity at the fiber tip was ~25–30 µW. GCaMP6_m_ fluorescence was recorded using the same objective, transmitted by the dichroic mirror filtered through a green fluorescence protein (GFP) bandpass emission filter (Thorlabs, Inc. Filter 525/39), and detected by the sensor of an CMOS camera (Thorlabs, Inc. DCC3240M).

A Labview program was developed to control the CMOS camera, which recorded calcium signals at 50 Hz. Behavioral event signals were recorded by a DAQ card (NI, usb-6001) at 1000 Hz using the same program.

### Single-unit and local field potential (LFP) recordings in freely moving mice

Both naive and chronically stressed mice (aged 8–12 weeks) were anesthetized with isoflurane (4.0% for induction and set-up on the animal bed, 0.8–1% during experiments) in a 20% O_2_/80% air mixture). Body temperature was maintained at 36–37 °C with a heating pad. For single-unit recording, mice were secured in a stereotaxic apparatus and a custom-made screw-driven microdrive containing eight tetrodes (four wires wound together) was unilaterally implanted in the left NAc shell; For LFP recordings, custom-made stereotrodes (two wires wound together) were unilaterally implanted in both left NAc shell and adBNST. Each stereotrode was housed in a silica tube and consisted of two individually insulated platinum-iridium wires (17 µm inner diameter). The electrodes were modified by electrochemical deposition of platinum to reduce their impedance to ~500 KΩ. The skull was leveled using bregma and lambda landmarks, and screws were implanted on the anterior and posterior portions of the skull to serve as reference. Coordinates were measured from bregma and depth was calculated from the brain surface. The electrodes were implanted through burr holes in the skull aimed at the following coordinates: AP 1.35 mm, ML 1.35 mm, and DV −4.85 mm for NAc shell and AP 0.20 mm, ML 0.80 mm, and DV −4.05 mm for adBNST. The microdrive electrode was attached to a micromanipulator and moved gradually to a position about 400 μm above the desired depth. The electrodes were anchored to the microdrive that made it possible to advance along the dorsal-ventral coordinates. Following surgery, mice were allowed to recover for at least one week and were then habituated to experimenter handling. During recording, electrodes were connected to a unitary gain headstage (Plexon, Dallas, TX) connected to a 32-channel preamplifier (Plexon, Dallas, TX). Once mice were familiar with the recording set-up they were connected to the headstage preamplifier in their home cages for two daily sessions of 20 min each. Neurophysiological signals were recorded with a 64-channel Multichannel Acquisition Processor (Plexon, Dallas, TX) and mouse positions were tracked using an overhead camera (30 Hz). Wideband signals were recorded at 40 kHz and LFP signals were acquired at 1 kHz.

At the end of each experiment, each mouse was deeply anesthetized with 10% chloral hydrate (0.4 mg/kg) and transcardially perfused with PBS, then 4% paraformaldehyde (PFA) (wt/vol). Brains were dissected and postfixed at 4 °C in 4% PFA overnight. Brains were then frozen and cut into 40 μm coronal slices and mounted on slides. Recording sites were marked with electrolytic lesions prior to perfusion and examined under a microscope to confirm recording locations.

### Resting-state MRI

All mice were initially anesthetized with isoflurane (4.0% for induction and set-up on the animal bed, 0.8–1% during experiments) in a 20% O_2_/80% air mixture. Body temperature was maintained at 37 °C using warm water circuitry. MRI experiments were conducted using a Bruker Biospec70/20USR small animal MR system (Bruker BioSpin MRI, Ettlingen, Germany) operating at 300 MHz (7 T). Breathing rate, heart rate and blood oxygen saturation were monitored using a pulse oximeter positioned at the hind limb and a pressure-sensitive sensor under the mouse chest (MR-compatible Small Animal Monitoring & Gating System, SA Instruments, Inc.).

A planar receive-only surface coil with 20 mm diameter was used in combination with a detunable partial volume transmit coil (BrukerBioSpin MRI, Ettlingen, Germany). T2 anatomical reference scans in the coronal plane were acquired using a spin echo (Turbo-RARE) sequence: field of view (FOV) = 18 × 18 mm^2^, matrix dimension (MD) = 256 × 256, repetition time (TR) = 5000 ms, echo time (TE) = 12 ms, RARE factor = 8, number of averages (NA) = 4, spatial resolution = 0.0703125 × 0.0703125 × 0.8 mm^3^, 12 slices, no gap. Resting-state data sets were then acquired using single-shot gradient-echo EPI (Echo Planar Imaging) with TR 1000 ms and TE 16 ms. Twenty coronal slices (using the same procedure as the T2 anatomical images above) were recorded with a FOV of 1.8 × 1.8 mm^2^ and matrix size of 64 × 64, resulting in voxel dimensions of 0.28125 × 0.28125 × 0.8 mm^3^. Each resting-state fMRI dataset comprised 300 repetitions, resulting in a scanning time of 10 min 40 s each. The bandwidth used was 200 kHz (6250 Hz/voxel). Preprocessing was done using SPM12 (http://www.fil.ion.ucl.ac.uk/spm/software/spm12/) to eliminate head movement and image shift by co-registering with the anatomical image, and then Gaussian smoothing was performed for every slice to improve the signal-to-noise ratio. To estimate functional connectivity, 4–5 voxels in each bilateral NAc image were selected as ROI (seed points), using REST (http://restfmri.net/forum/index.php) and home-written algorithms using Matlab2014a (www.mathworks.com). For the chronically stressed group, MRI was conducted 48 h after the last stressor. Differences in functional connectivity between the stressed and naive groups were calculated by subtraction of the functional connectivity values between the stressed and naive groups, and statistically significant differences were tested using a two-sample *t*-test.

### Stereotactic virus injection and optogenetic manipulation

Cre-mice (6–12 weeks) were used for stereotactic viral injections in the NAc shell. During isoflurane anesthesia (as above), the skull was exposed via small incision and a small hole was drilled for injection. A modified microliter syringe (Hamilton) with a 22-gauge needle was used: the tip of the needle was placed at the target region and the injection was performed at a speed of 100 nl/min using a micromanipulator (for coordinates and volumes see below). The needle was left in place for 10 min after injection. Viral injections were unilateral for optogenetic, fiber photometry, slice-physiology connectivity and rabies mapping experiments. For DREADD and taCasp3 experiments the viral injections were bilateral. For optogenetic and fiber photometry experiments, mice were also implanted with a unilateral fiber-optic cannula secured to the skull with dental cement. Fiber-optic cannulas were 200 μm for optogenetic and fiber photometry experiments. We used the following stereotactic coordinates (in mm): NAc (AP +1.35, ML ±1.35, DV −5.05 (virus) and –4.85 (fiber optic)), adBNST (AP +0.20, ML +0.80, DV –4.05 (virus)). Adeno-associated viruses (AAVs) carrying Cre-inducible (double-inverse orientation; DIO) transgenes were packaged in our laboratory (AAVs for optogenetics, DREADD, taCasp3) or purchased from BrainVTA (Wuhan, China. http://brainvta.com) (AAVs for retrograde tracing or fiber photometry). Glycoproteindeleted rabies virus for retrograde tracing (RV-ENVA-ΔG-dsRed 2.0 × 10^8^ IFU/mL) was also purchased from BrainVTA.

### In vivo anesthetized electrophysiology

Adult mice (8–12 weeks) were anesthetized with isoflurane (4.0% for induction and set-up on the animal bed, 0.8–1% during experiments) in a 20% O_2_/80% air mixture). Once anesthetized, mice were placed into a stereotactic frame and body temperature was maintained at ~37 °C using a heating pad. A recording electrode was implanted into the NAc shell and a reference electrode was implanted in the contralateral NAc shell. Optical stimulation-induced neuronal activity was measured by calculating the firing rate 10 s before the stimulus and 40 s during the stimulus and the time bin of the firing rate was set to 500 ms. To determine when a single unit significantly responded to the optical stimulus, we used the criterion that the unit’s *P* value of the ranksum test needed to be less than 0.05. The average firing rate of each group of neurons was calculated after normalizing the firing rate of each unit by Z-score method. To further examine the optical response for the classified neurons, optical-induced firing probabilities were also calculated with the parameters of 15 ms pre-time, 15 ms post-time, and 0.1 ms time bin.

### Patch-clamp electrophysiology

Coronal slices (300 μM) containing NAc shell (bregma 1.7–0.6 mm) were prepared from *PV-Cre* transgenic mice using standard procedures. Brains were quickly removed and chilled in ice-cold modified artificial cerebrospinal fluid (ACSF) containing (in mM): 110 Choline Chloride, 2.5 KCl, 1.3 NaH_2_PO_4_, 25 NaHCO_3_, 1.3 Na-Ascorbate, 0.6 Na-Pyruvate, 10 Glucose, 2 CaCl_2_, 1.3 MgCl_2_. NAc slices were then cut in ice-cold modified ACSF using a Leica vibroslicer (VT-1200S). Slices were allowed to recover for 30 min in a storage chamber containing regular ACSF at 32~34 °C (in mM): 125 NaCl, 2.5 KCl, 1.3 NaH_2_PO_4_, 25NaHCO_3_, 1.3 Na-Ascorbate, 0.6 Na-Pyruvate, 10 Glucose, 2 CaCl_2_, 1.3 MgCl_2_ (pH 7.3~7.4 when saturated with 95% O_2_/5% CO_2_), and thereafter kept at ~25 °C until placed in the recording chamber. The osmolarity of all the solutions was 300~320 mOsm/Kg.

For all electrophysiological experiments, slices were viewed using infrared optics under an upright microscope (Eclipse FN1, Nikon Instruments) with a 40x water-immersion objective. The recording chamber was continuously perfused with oxygenated ACSF (2 ml/min) at 34 °C. Pipettes were pulled by a micropipette puller (Sutter P-2000 Micropipette Puller) with a resistance of 3–5 MΩ. Recordings were made with electrodes filled with intracellular solution (in mM): 130 potassium gluconate, 1 EGTA, 10 NaCl, 10 HEPES, 2 MgCl_2_, 0.133 CaCl_2_, 3.5 Mg-ATP, 1 Na-GTP. Inhibitory postsynaptic potentials (IPSPs) were recorded with PV cells held at 30 pA and the recording electrodes (7–9 MΩ) were filled with a solution containing (in mM): 120 cesium methansulphonate, 20 HEPES, 0.4 EGTA, 2.8 NaCl, 5 tetraethylammonium chloride, 2.5 MgATP, 0.25 NaGTP (pH 7.4, 285 mOsm/kg). AP-5 (25 μM) and NBQX (25 μM) were added to the ACSF during IPSP recording. IPSPs were blocked by adding 20 μM bicuculline (GABA_A_ receptor antagonist). Action potential firing frequency was analyzed in current-clamp mode in response to a 2 s depolarizing current step. Rheobase was determined as the amplitude of a minimum current step (advanced in 10 pA increments) to elicit an action potential response. Paired-pulse ratio (PPR) was recorded in current-clamp mode by giving two pulse stimulation with the interval of 50 ms, 100 ms, 150 ms and 200 ms. All recordings were conducted with a MultiClamp700B amplifier (Molecular Devices). Currents were low-pass filtered at 2 kHz and digitized at 20 kHz using an Axon Digidata 1440 A data acquisition system and pClamp 10 software (both from Molecular Devices). Series resistance (Rs) was 10~30 MΩ and regularly monitored throughout the recordings. Data were discarded if Rs changed by >25% over the course of data acquisition.

### Measurements of norepherine (NE) and Corticotropin-releasing hormone (CRH)

Blood samples were collected from naive and stressed mice. Serum was prepared after each blood sample was centrifuged at 1000 rpm. Serum aliquots were immediately frozen at −80 °C prior to being used. NE and CRH in the serum were determined using the Radioimmunoassay Kits (Xinfan Biotechnology Co., Ltd, Shanghai, China) in accordance with the manufacturer’s instructions.

### Histology and confocal microscopy

Mice were deeply anesthetized and transcardially perfused with ice-cold 4% paraformaldehyde (PFA) in PBS (pH 7.4). Brains were fixed overnight in 4% PFA solution and then equilibrated in 30% sucrose in PBS. Next, 30 μm coronal slices were cut using a freezing microtome. Slices were stored in a cryoprotection solution at 4 °C until further processed. The sections were incubated with primary antibodies overnight at 4 °C. Alexa Fluor^®^488, 594 or 647-conjugated goat anti-rabbit or anti-mouse IgG antibodies or anti-chicken IgY antibodies (1:500; Invitrogen, CA, USA) were the secondary antibodies. Nuclei were counterstained using DAPI. Immunostaining was performed using mouse anti-parvalbumin (1:300, Millipore, MA, USA) and rabbit anti-choline acetyltranserase (1:200, Abcam, ab6168). Histological slides were imaged on a (Zeiss LSM880) confocal microscope using a 10×, 20× or 63× objective.

### Retrogradely monosynaptic tracing

For monosynaptic retrograde tracing, 100 nl of mixed helper AAV (Ef1a-DIO-His-EGFP-2a-TVA-WPRE-pA and Ef1α-DIO-RVG-WPRE-pA) (BrainVTA) was injected into the NAc shell of PV-Cre transgenic mice using coordinates of +1.35 AP, +1.35 ML, and −5.05 DV. Three weeks later, rabies virus EnvA-pseudotyped RV-ΔG-DsRed (200 nl) (BrainVTA) was injected into the NAc shell using the same coordinates. Approximately 7 days after the second injection, mice were anaesthetized with 10% chloral hydrate (0.4 mg/kg) and transcardially perfused with PBS, then 4% PFA (wt/vol) and then brain slices were prepared for tracing with dsRed.

### Fluorescence in situ hybridization

We used single-probe or double in situ hybridization (ISH) on fixed-frozen sections. For single-probe, coding region fragments of Gad1, Gad2, Vglut1, Vglut2, SOM, DRD1, DRD2, PV and CRH were isolated from mouse brain cDNA using PCR and cloned into the pCR4 Topo vector (Thermo Fisher). Digoxigenin (DIG)-labeled riboprobes were prepared using a DIG RNA Labeling Kit (11277073910, Roche). Brain sections were hybridized to DIG-labeled cRNA probes at 56 °C for 14–16 h. After hybridization, sections were washed twice in 0.2× SSC at 65 °C for 20 min and then incubated with horseradish peroxidase (POD)-conjugated sheep anti-DIG antibodies (1:300; 1207733910, Roche) diluted in blocking buffer (1% Blocking reagent, FP1012, Perkin Elmer) for 45 min at room temperature (RT). Sections were washed three times for 5 min at RT in PBST (0.05% Tween 20 in 1 X PBS) wash buffer, and then treated using a TSA-plus Cy5 kit (1:100; NEL745001KT, Perkin Elmer) for 10 min at RT. For double in situ hybridization using the RNAscope assay, we used probes for SST (Mm-SST-C1 probe), CRH (Mm-CRH-C2 probe, 1:50 dilution) and PDYN (Mm-PDYN-C2 probe, 1:50 dilution). All probes were purchased from ACD (Advanced Cell Diagnostics). In situ hybridization was performed on fixed-frozen sections according to the manufacturer’s protocol. Sections were washed two times for 5 min at RT in PBST and then incubated with Anti-RFP antibody (1:200; ab62341, Abcam) or anti-GFP (1:200; ab13970, Abcam) for 1.5 h at RT, and washed. Sections were incubated with Alexa Fluor 594-conjugated goat anti-rabbit IgG antibodies (1:200; 115-587-003, Jackson Immuno Research) for 1 h at RT. Sections were mounted in Fluoromount-G (0100-20, Southern Biotech) and then imaged using LSM 880 confocal microscopes (Zeiss, LSM880).

### Statistical analysis

No statistical methods were used to predetermine sample size. The sample size was chosen based on published studies in this field. The animals with incorrect electrodes placements, virus mis-injections, and incorrect optic fiber placements were excluded from our analysis. All statistical parameters for specific analyses are described in the appropriate figure legends. All data are presented as mean values ±S.E.M. Statistical significance was assessed by one or two-tailed Student’s *t* tests, two-sample *t*-test by using Graphpad software. Differences with *p* < 0.05 were considered statistically significant.

### EPM score

ANOVAs were used to identify neurons regulated by the EPM arm types and were calculated using the firing rate of each neuron with a three-level factor (closed arms, open arms and center zone) after each neuron’s spike train was binned into 3 s bins [27]. A neuron’s firing rate was considered to be influenced by EPM position if the rate in one maze area was statistically significantly higher than that in the others (closed arms versus open arms and center zone, open arms versus closed arms and center zone, center zone versus closed arms and open arms, Bonferroni post hoc test, ***P < 0.001) [27]. EPM scores were used to quantify the degree to which a neuron can distinguish the structure of the EPM [27, 28]; EPM scores were calculated as previously described [28, 29].

Score = (A − B)/(A + B), where

A = 0.25 × (|F_L_ − F_U_| + |F_L_ − F_D_| + |F_R_ − F_U_| + |F_R_ − F_D_|) and

B = 0.5 × (|F_L_ − F_R_| + |F_U_ − F_D_|).

Horizontal and vertical arms represent closed and open arms, respectively. F_R_, F_L_, F_D_ and F_U_ are the % differences from mean firing rate in right, left, down and up arms, respectively; A is the mean difference in normalized firing rate between different-type arms and B is the mean difference for same-type arms. Neurons with firing patterns related to the EPM task have a high EPM score, as neurons will have similar firing rates in the same arm types (resulting in a small B value) and large differences in rates between different arm types (resulting in a large A value). The maximum EPM score of 1.0 shows no difference in firing rate across arms of the same type (B = 0). Negative EPM scores show that firing rates were more similar across arms of different types than across arms of the same type.

We calculated whether there was a statistically significant difference between the population of experimentally observed EPM scores from that expected by chance using a bootstrapping method. For each unit that had n spikes, 500 simulated EPM scores were generated by calculating the EPM score of n randomly chosen time stamps 500 times. 500 × 98 EPM scores were generated from 98 units recorded. Statistical differences between experimentally observed EPM scores of all neurons and chance were calculated by comparison to the simulated distribution of EPM scores using the Wilcoxon rank-sum test [27, 28].

The firing pattern of NAc neurons at transitions between different types of EPM arms and Z-scores of firing rate were calculated for each unit for 10 s and averaged over total transitions for each unit. We identified a point where there was a change in the slope of the averaged z-scores. The averaged z-scores were divided into two parts by using this identified change point. Since these data did not follow a normal distribution and did not obey homogeneity of variance, a nonparametric Kolmogorov–Smirnov test was used to evaluate whether there were statistically significance differences between the means from these two data segments. This was calculated using 0.25 s bins.

### In vivo calcium signal analysis

Within each heat map, every row was normalized from 0 to 1 according to the formula (*D* − *D*_min_)/(*D*_max_ − *D*_min_), where *D* is the raw fiber photometry signal data, *D*_min_ is the minimum value of a given row and *D*_max_ is the maximum value of the same row. We sorted every row according to the time of peak value from late to early. To compact the heat map, we inserted one thousand data points between every two points of raw data by applying cubic spline methods. The raw heatmap data were normalized by Z-Score normalization. The formula for Z-Score is (*D* − *μ*)/*σ*, where *D* is the raw fiber photometry signal data, *μ* is the mean value of raw data and *σ* is the standard deviation of raw data. The Z-Score data was divided into two even parts by time zero (defined by the time that mouse moved from closed arm to center zone). To visualize the mean recording traces from the activity of different NAc neuronal populations in the EPM, the first three seconds was chosen as a baseline and then Z-Score normalization was applied to all data using the Δ*F*/*F* = (*F* *−* *F*_*0*_)*/F*_*0*_ method, where *F* is the normalized fiber photometry signal data and *F*_*0*_ is the mean normalized data value. All calculations were performed using MATLAB 2017a, GraphPad Prism7 and SPSS 18.

### Single-unit spike sorting and analysis

Single-unit spike sorting was performed using Offline sorter (Plexon). Wideband signals were high-pass filtered (300 Hz) with a Bessel filter for detection of the spikes. The threshold value for spike detection was −4.5 standard deviations and spike waveforms were recorded for a time window of 1400 μs starting 300 μs before threshold crossing. Principal-component values were calculated for the unsorted aligned waveforms and plotted in three-dimensional principal-component space. A group of waveforms was considered to be generated from one single unit if the waveforms were distinct from other clusters in the principal-component space and exhibited a refractory period more than 1 ms. In order to avoid analysis of the same neuron on different channels, cross-correlation histograms were calculated: if a neuron showed a peak at the same time as the reference neuron fired, only one of the two neurons was reserved for further analysis. To quantify the separation between identified neurons, L ratio and Isolation Distance [30] were calculated. High values of the Isolation Distance and low values of the L ratio indicated good cluster separation. The L ratio estimates the degree of noise contamination of one cluster, and a smaller value implied a lower degree of contamination. The Isolation Distance measured the average distance expected between a cluster and an equal ensemble of spikes outside the cluster, and a bigger value indicated a well-isolated cluster. The threshold values of the L ratio and Isolation Distance were set to 0.2 and 15, respectively [31]. Units with L ratio higher than 0.2 and Isolation Distance lower than 15 were excluded from the following analysis. Classification of NAc neurons were as described in previous studies and two features used for this, peak-to-peak width and average firing rate, were calculated for each unit [32, 33]. An unsupervised cluster algorithm based on Ward’s method was used to classify the neurons. Euclidian distance was calculated between neuron pairs based on the two-dimensional space defined by the two features [34]. To calculate a neuron’s burst number, a burst was defined as comprising at least three spikes with interspike intervals < 9 ms [35]. Neuron firing rates were considered as having undergone a statistically significant change if the *P* value of the *ranksum* test was less than 0.05. The multitaper method [36] in the Chronux analysis package (http://chronux.org) was used for power spectra, time frequency and coherence analysis. The value was calculated using a 1 s window, 3 time-bandwidth product (NW) and 5 tapers. The coherence value in the *theta* band (4–12 Hz) that exceeded the 95% confidence level was used for the analysis. The coherence value was normalized by dividing by the maximum value in the *theta* band. The statistical analysis of the coherence was conducted on the original values.

## Results

### Chronic stressors increase functional brain connectivity between the BNST and NAc

**Fig. 1:**
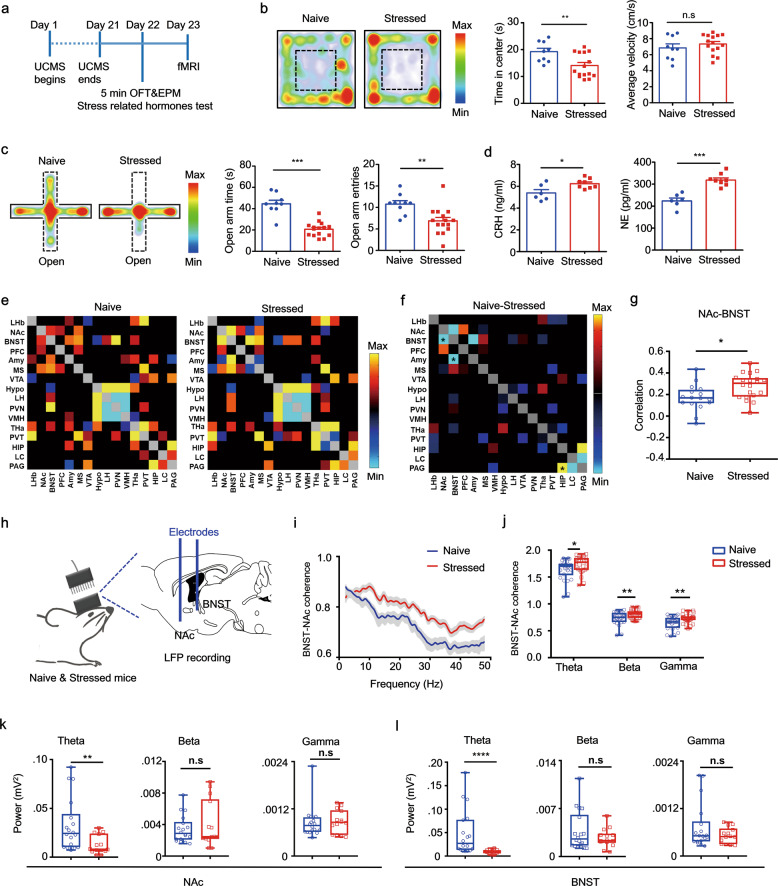
Chronically stressed mice exhibited increased functional brain connectivity in the BNST and NAc. **a** Protocol used for unpredictable chronic mild stress (UCMS) and functional brain connectivity measurement and paradigm for behavioral assay. **b** Left, representative mouse trajectory map for both naive (*n* = 9 mice) and stressed groups (*n* = 15 mice) in the open field test (OFT), warm colors represent high time spent; middle, time spent in central compartment; right, average velocity in the OFT (Unpaired *t* test, middle, *t* = 2.401, *P* = 0.0241; right, *t* = 1.591, *P* = 0.1241). **c** Left, representative mouse trajectory map in the elevated plus maze (EPM), middle, time spent in the open arms; right, open-arm entries; (Unpaired *t* test, middle, *t* = 7.145, *P* < 0.0001; right, *t* = 3.214, *P* = 0.0040). **d** *Left* serum CRH and *right* NE concentrations of naive control (*n* = 6 mice) and stressed groups (*n* = 8 mice) (Unpaired *t* test, *left*, *t* = 2.671, *P* = 0.0204; *right*, *t* = 5.865, *P* < 0.0001). **e** Correlation matrices derived from global fMRI BOLD signal analysis (pseudocolor map of *t* statistics after thresholding at a false discovery rate, *P* of 0.05) across brain regions in stressed compared with wild-type naive littermates; warm colors represent higher correlations. **f** Difference in functional connectivity between stressed and naive littermate groups. Red, naive > stress; blue, naive < stress, *significant difference (*t* test, BNST-NAc: *t* = −2.69812, *P* = 0.010777; BNST-Amy: *t* = −3.14356, *P* = 0.003453; PAG-HIP: *t* = 2.315667, *P* = 0.026741). **g** Correlation of BOLD synchronization in BNST-NAc in stressed (*n* = 7 mice) and naive littermates (*n* = 5 mice) (Unpaired *t* test, *t* = 2.698, *P* = 0.0108). **h** Schematic showing local field potential recording strategy. **i** Coherence between LFP signals recorded from NAc and BNST in stressed (*n* = 7 mice) and naive control mice (*n* = 7 mice). **j** Coherence in *theta* (4–12 Hz), *beta* (12–30 Hz) and *low gamma* (30–50 Hz) bands in NAc and BNST (Unpaired *t* test, *theta*: *t* = 2.065, *P* = 0.0440, *beta*: *t* = 2.584, *P* = 0.0126, *low gamma*: *t* = 2.856, *P* = 0.0061). **k**–**l** Sum of power spectra obtained for LFP recordings in NAc shell (Mann–Whitney rank sum test, *theta*: *P* = 0.0058, *beta*: *P* = 0.8662, *low gamma*: *P* = 0.6395) and BNST (Mann–Whitney rank sum test, *theta*: *P* = 0.004, *beta*: *P* = 0.5867, *low gamma*: *P* = 0.5867). *P < 0.05, **P < 0.01 and ***P < 0.0001. Error bars (**c**, **d**, **g**, **j**–**l**) are mean ± SEM. In **i**, the curves and shaded areas indicate the mean ± SEM.

To gain a circuit-level understanding of anxiety-like behavior, we adopted a chronic stress model to investigate specific brain regions in the regulation of avoidance behavior under anxious states. After exposure to chronic stressors (Fig. 1a), mice showed higher anxious states by markedly avoiding the center in open-field test (OFT) (Fig. 1b left and middle) and open-arms of an elevated plus maze (EPM) (Fig. 1c) when compared with their naive littermates. Locomotor activity in stressed mice was not significantly different to that of their naive littermates (Fig. 1b, right). Stress-related hormones, corticotropin-releasing hormone (CRH) and norepinephrine (NE), were both significantly higher in chronically stressed mice (Fig. 1d). These data indicate that chronic stressors disrupted normal anxiety-like behavior and resulted in a maladaptive and excessive avoidance coping response.

We further tested global functional brain connectivity by quantifying the synchronization of blood oxygen level-dependent (BOLD) fMRI signals across brain regions in anesthetized naive and chronically stressed mice (Fig. 1e). The synchronization of BOLD signals in the BNST and NAc was significantly higher in chronically stressed mice compared with their naive littermates (Fig. 1g). Subtraction of functional connectivity values between the stressed and naive groups showed a significant difference between the BNST and NAc, and between the BNST and amygdala (Amy), hippocampus (HIP) and periaqueductal gray (PAG) (Fig. 1f). Moreover, functional connectivity was higher in stressed mice compared with their naive littermates between the basolateral amygdala (BLA) and BNST, and lower between the PAG and HIP (Fig. S1a, left and middle), whereas connectivity between the NAc and PFC was not significantly different between groups (Fig. S1a, right). Consistent with the fMRI synchronization data, fMRI heat maps in coronal sections generated from stressed mice show a higher correlation of resting-state fMRI BOLD signal than those generated from their naive littermates, with a seed in NAc across brain regions (Fig. S1b, top) between BNST and NAc (Fig. S1b, bottom).

To quantify long-range functional connectivity, we measured local field potential (LFP) coherence between the BNST and NAc in awake behaving stressed and naive mice (Fig. 1h). Compared with naive littermates, the stressed mice showed higher BNST-NAc coherence in *theta*, *beta*, and *low gamma* bands (Fig. 1i, j). We then analyzed the local *theta*, *beta*, *low gamma* rhythms, respectively, in these two brain regions and found that local *theta* power was significantly lower in stressed mice compared with their naive littermates in both structures, whilst there was a trend towards lower *gamma* power in stressed mice in the BNST (Fig. 1k, l).

Since in the cortex and hippocampus, changes either in *theta* [37, 38] or in *gamma* power [39] is due to the PV cell functions, we then investigated PV neuronal firing features within the BNST-NAc circuit in chronically stressed mice.

### Chronically stressed mice have higher sNAc^PV^ neuron firing rates

**Fig. 2:**
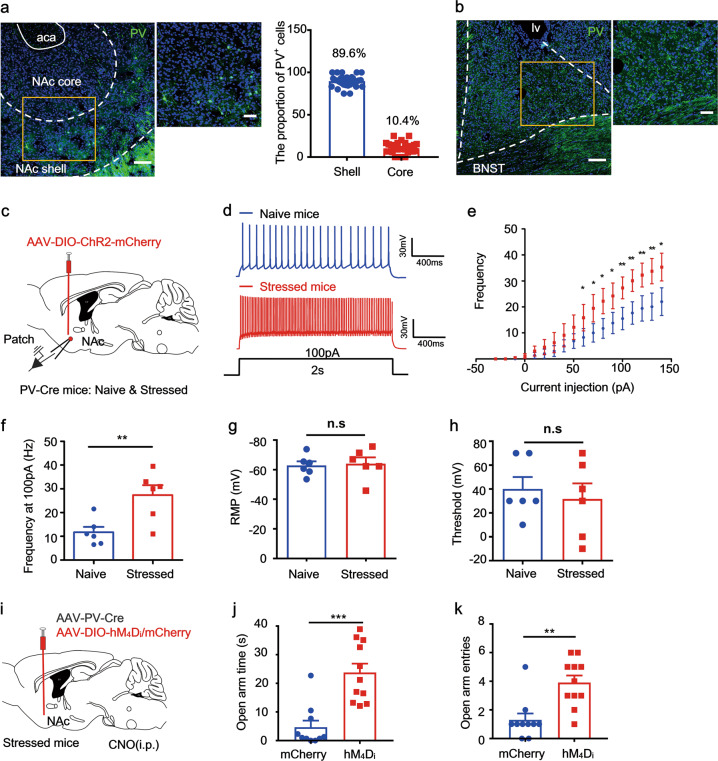
Accumbal PV neurons exhibit higher excitability in chronically stressed mice, and are responsible for excessive avoidance of anxiogenic locations. **a** Representative images showing the distribution of PV neurons (green) in the NAc; *left*, scale bar, 100 μm; *middle*, scale bar, 50 μm; *right*, the proportion of PV neurons in the NAc shell and core area, respectively (*n* = 31 slices from 3 mice). **b** Representative images showing the distribution of PV neurons (green) in BNST; *left*, scale bar, 100 μm; *right*, scale bar, 50 μm. **c** Schematic showing selective expression of ChR2 into the sNAc^PV^ neurons for whole-cell patch-clamp recordings. **d** Representative traces showing action potential spiking in sNAc^PV^ neurons (*n* = 6 cells from 4 mice per group). **e** The input–output curve of injected currents versus spiking frequency. **f** Spiking frequency of PV neurons in naive and stressed mice during the injection of a current (100 pA, unpaired *t* test, *t* = 3.284, *P* = 0.0082). **g**–**h** RMP and threshold of naive and stressed mice (Unpaired *t* test, for RMP, *t* = 0.2418, *P* = 0.8139; for threshold, *t* = 0.5077, *P* = 0.6227). **i** Schematic showing chemogenetic inhibition of sNAc^PV^ neurons. **j**–**k** Mean time spent in open arms and entries to the open arms with or without chemogenetic inhibition of sNAc^PV^ neurons (*n* = 10–11 mice per group, unpaired *t* test, for **j**, *t* = 4.852, *P* = 0.0001; for **k**, *t* = 3.880, *P* = 0.001).

Next, we looked at the distribution of PV neurons in both the BNST and NAc and found that PV neurons were expressed predominantly in the NAc shell (sNAc, Fig. 2a, Shell vs. Core, 89.6% vs. 10.4%, Fig. 2a, right). Consistent with other work [40], we found no expression of PV soma in the BNST, only terminal structures (Fig. 2b). To further investigate the causal relationship of PV neuronal activity and excessive avoidance observed in stressed mice, we tested sNAc^PV^ neuronal firing properties in stressed mice. We selectively expressed ChR2-mCherry in sNAc^PV^ cells of PV-Cre mice to visualize the PV neurons and recorded their firing patterns in response to electrical stimulation (Fig. 2c). PV neurons in stressed mice exhibited an increase in firing frequency in response to injection currents (Fig. 2d–f) without changes in resting membrane potential (RMP) or threshold (Fig. 2g, h). If excessive avoidance in stressed mice is due to increased excitability of the sNAc^PV^ neurons, it may be possible to rescue this maladaptive behavior by inhibiting sNAc^PV^ neuronal activity. To test this possibility, we injected AAV-PV-Cre and either DIO-hM_4_D_i_ or DIO-mCherry into the sNAc of stressed mice (Fig. 2i). Compared with the mCherry group, chemogenetic inhibition of PV neurons rescued the excessive avoidance effect observed on the open arms of EPM, as seen in both open arm time and entries, which were significantly higher (Fig. 2j, k). These results imply that hyper-excited sNAc^PV^ neurons in stressed mice contributed to their excessive avoidance behavior.

### PV neurons in sNAc represent an anxiety-like signal and impact avoidance coping behavior

**Fig. 3:**
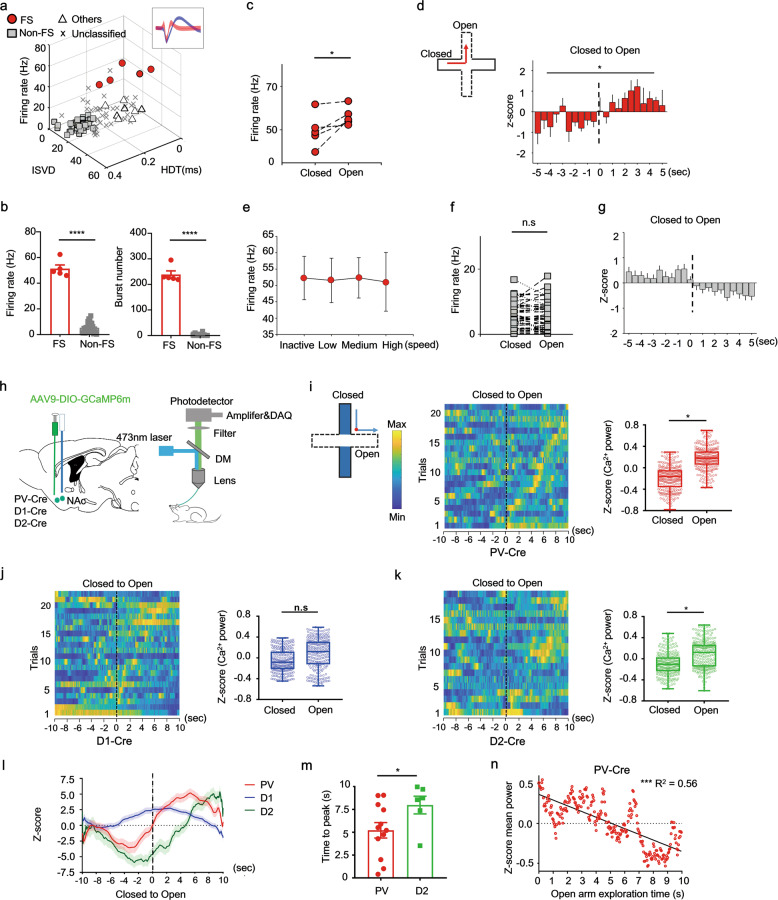
Different neuronal populations in the NAc shell show distinct firing preference before and during exploration of anxiogentic environments. **a** Scatter plot of the firing rate and peak-to-peak width for 98 units from 9 mice. *Inset*, representative waveforms from an identified Non-FS (blue) and FS (red) neuron; ISVD, initial slope of valley decay; HDT, valley half decay time. **b** *Left*, mean firing rates of FS and Non-FS neurons (Mann–Whitney rank sum test, *n* of FS = 5, *n* of Non-FS = 28, *P* <= 0.0001); *right*, burst number of FS and Non-FS neurons (Mann–Whitney rank sum test, *n* = 5 (FS), *n* = 28 (Non-FS), *P* <= 0.001). **c** Scatter plot showing the firing rate of FS neurons between closed and open arms (Paired *t* test, *n* = 5, *t* = 2.135, one-tailed *P* = 0.0498). **d** Z-scores of FS neurons during movement from the closed to open arms (nonparametric Kolmogorov–Smirnov test, *n* = 5, *P* = 0.0301); *inset*, horizontal and vertical arms represent closed and open arms, respectively. **e** Correlation between locomotion and firing rates of the FS neurons. **f** Scatter plot showing the firing rates of Non-FS neurons in closed and open EPM arms (Paired *t* test, *n* = 28, *t* = 0.64, one-tailed *P* = 0.2619). **g** There was no difference in individual Non-FS neuron firing rates between closed and open arms. **h** Schematic showing fiber photometry recording strategy. **i**–**k** *Left*, normalized NAc Ca^2+^ activity maps from different neuron populations in the EPM (warm colors, high activity), binned by time (s) from the EPM crossing point (*inset*, red point); *inset*, horizontal and vertical arms represent open and closed arms, respectively; *right*, normalized NAc Ca^2+^ transients of different neuron populations in the EPM open arms compared with closed arms (Wilcoxon test, *n* = 250, *P < 0.05). **l** Mean activity traces from different NAc neuron populations during the EPM. **m** Comparison of the time to peak between PV and D2 cells when mice were approaching the crossing point in the EPM (*n* = 19 trials, *t* = 2.149, one-tailed *P* = 0.0261). **n** Normalized Ca^2+^ transients in NAc PV neurons was correlated with anxiety state (Open arm exploration time) (linear regression, F (1, 248) = 316.4, R^2^ = 0.56, *P* < 0.0001, *n* = 8 mice).

We next investigated how NAc neurons are engaged in anxiogenic information processing under physiological conditions using a combination of single-unit and photometry recordings in freely moving mice. These approaches allowed us to record neuronal firing events in the NAc shell whilst mice freely explored safe/threatening environments. Ninety-eight well-isolated NAc units from nine mice during the EPM assay were recorded. Distinct sub-types of NAc neurons were classified based on their major electrophysiological properties [32, 41]. Neurons were classified as: (1) putative fast-spiking units (FS) if mean firing rate was more than 15 Hz, the initial slope of valley decay was greater than 22 mv/μs and the valley half decay time was less than 250 μs; (2) putative non-fast spiking units (Non-FS) if the firing rate was less than 2 Hz, the initial slope of valley decay was less than 22 mv/μs, and the valley half decay time was greater than 250 μs; (3) others (units that could not be classified as FS or non-FS) or (4) unclassified, the units could not be identified as neurons (Fig. 3a). A total of five putative FS neurons (5.1%) and 28 putative Non-FS neurons (28.6%) were clearly identified (Fig. 3a). These two classes of neurons had significantly different mean firing rates and burst numbers (Fig. 3b). Individual FS units showed a firing preference for the open arms over the closed arms (Fig. 3c). The z-scores of FS unit firing rates increased when mice entered an open arm (Fig. 3d) and decreased when they moved to closed arms (Fig. S2a). Note that FS unit firing rates were not influenced by locomotion speed (Fig. 3e). By contrast, the individual Non-FS units showed no firing preference for either closed or open arms (Fig. 3f) and none of the z-scores of the firing rates were modulated by movement over the crossing point in the EPM (Fig. 3g, and see also Fig. S2b). We further checked the local *theta* power (4–12 Hz) during exploration of either closed or open arms and found *theta* power was significantly lower during exploration of open arms compared with closed arms, implying an anxious state (Fig. S2c). There was no difference in *theta* power at different locomotion speeds (Fig. S2d). To show the temporal dynamics of the NAc *theta* oscillation on the EPM, a mean time-frequency map of mice traversing between arms was calculated and aligned to entrance time points. The map shows a decline in mean *theta* power when mice left the closed arms (Fig. S2e, *left*, indicated by the black dotted line), which increased when they returned to the closed arms (Fig. S2e, *right*, indicated by the black dotted line). Finally, to further address the relationship between local spike activity and neural oscillations, we calculated the mean spike-field coherence for both FS and Non-FS units. We found that FS, but not Non-FS, had strong coherence between their spikes and *theta* oscillations at 4–12 Hz (Fig. S2f). These results imply that NAc FS activity was inversely correlated with *theta* power, and that a reduction of the accumbal *theta* activity reflects a higher stress load during exploration of the threatening environments, which can promote adaptive avoidance behavior. These findings are consistent with the above result showing that a decrease in local *theta* power within either NAc or BNST is reflected by the anxious state of the stressed mouse (see also Fig. 1j, k).

PV neurons are fast-spiking neurons [42–44], and D1, D2-MSNs are the dominant non-fast spiking cell types in the NAc [45]. We next used in vivo calcium signal recordings in three different strains of transgenic mice to confirm the impact of these different neuron types on anxiogenic information processing (Fig. 3h). We first performed Immunostaining and *in situ* hybridization and confirmed the specific expression of GCaMP6_m_ in PV, D1R or D2R cells respectively (Fig. S3a–c). Ca^2+^ signals were then recorded as mice moved from the closed to open arms (Fig. 3i, inset). PV neurons within sNAc were activated when mice approached the boundary between the closed and open arms and Ca^2+^ signal increased significantly during exploration of the open arms (Fig. 3i); D1 MSNs exhibited no preference for either closed or open arms (Fig. 3j) whereas D2 MSNs showed a slightly different firing preference for the open arms to the closed ones (Fig. 3k). Recording traces from the different populations of NAc neurons were used to generate z-scores of calcium signal change and we found that PV and D2 neurons were active when mice were in the open arms but not the closed arms (Fig. 3l); however, the time to peak of D2 neuronal firing occurred significantly later than PV neurons during exploration of the open arms (Fig. 3m). These neuronal firing patterns in the open arms reflect that sNAc^PV^ neurons have an important impact on anxiety-like avoidance behavior. In addition, we found a negative correlation between the PV firing rates and time of open-arm exploration (Fig. 3n), which suggests that accumbal PV neuronal activation plays a role in avoidance of anxiogenic locations. These response profiles were absent in control mice expressing eYFP (Fig. S3d–f), indicating the GCaMP6_m_ signal was not a locomotion artifact.

### Activation of PV neurons in NAc shell is required for avoidance coping responses to anxiogenic stimuli

**Fig. 4:**
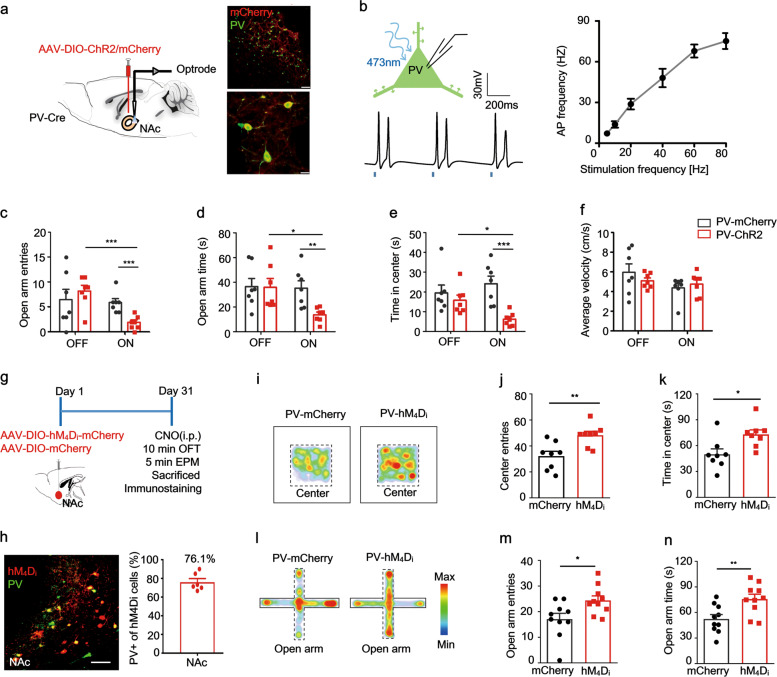
Activation of PV neurons in sNAc is required for anxiety-like avoidance. **a** *Left*, Schematic showing optrode placement in the NAc shell; *Top right*, ChR2-mCherry neurons (red) in NAc co-expressed PV (green); scale bar, 100 μm; *bottom right*, higher magnification of the co-staining (merged, yellow; scale bar, 10 μm). **b** *Top left*, schematic showing patch-clamp technique; *bottom left*, sample of light-evoked PV neuronal action potentials; *right*, the action potential frequency of PV neurons following different light stimulation frequency from 5–80 Hz. Comparison of **c** open arm entries and **d** time spent in the open arm between PV-ChR2 and PV-mCherry groups (*n* = 7 per group, unpaired *t* test, for c, ChR2: OFF vs. ON, *t* = 4.950, *P* = 0.0003; ON: mCherry vs. ChR2, *t* = 4.338, *P* = 0.001; for **d**, ChR2: OFF vs. ON, *t* = 3.024, *P* = 0.0106; ON: mCherry vs. ChR2, *t* = 3.383, *P* = 0.0054). **e** Comparison of time spent in the central area during OFT between PV-ChR2 and PV-mCherry groups (*n* = 7 per group, unpaired *t* test, ChR2: OFF vs. ON, *t* = 2.661, *P* = 0.0207; ON: mCherry vs. ChR2, *t* = 4.447, *P* = 0.0008). **f** Mean velocity of PV-ChR2 and PV-mCherry groups during OFT (*n* = 7 per group, unpaired *t* test, ChR2: OFF vs. ON, *t* = 0.6542, *P* = 0.5253; ON: mCherry vs. ChR2, *t* = 0.6205, *P* = 0.5466). **g** Protocol schematic for selective chemogenetic inhibition of PV neurons in the sNAc. **h** *Left*, immunostaining showing targeted hM_4_D_i_ expression (red) in PV neurons (green) of sNAc in PV-Cre mice; scale bar, 50 μm. *Right*, quantification of PV cells with hMeDi expression, *n* = 6 slices from 3 mice. **i** Representative mice thermal tracks for *left* PV-mCherry and *right* PV- hM_4_D_i_ groups during OFT. **j**–**k** Mean number of entries to center (*n* = 8 mice per group, unpaired *t* test, *t* = 3.357, *P* = 0.0047) and mean time in the center (Unpaired *t* test, *t* = 2.805, *P* = 0.0140). **l** Representative mice thermal tracks for *left* PV-mCherry and *right* PV- hM_4_D_i_ groups during EPM task. **m**, **n** Mean number of open arm entries (*n* = 10 mice per group, unpaired *t* test, *t* = 2.571, *P* = 0.0192) and mean time in open arms (Unpaired *t* test, *t* = 3.042, *P* = 0.0070).

A combination of PV-Cre mice, conditional ChR2 viral expression, and optogenetic manipulation was used to test the impact of accumbal PV neuronal activity on avoidance behavior induced by an anxiogenic context. Because fast-firing PV cells can generate high-frequency trains with maximal frequencies greater than 100 Hz, and above 60–80 Hz the slope of frequency-interval (f-I) curve is well-approximated by linearization [46], we used a photostimulation rate of 60 Hz for the optrode placed in the NAc shell (Fig. 4a, *left*); this is approximately the average firing rate of FS units in the open arms (see also Fig. 3c). Immunostaining results indicated that the majority of ChR2-mCherry labeled neurons expressed PV (Fig. 4a, *right*). We confirmed that opto-tagged PV^+^ cells, recorded from patch-clamp experiments in the acute brain slices, were steadily activated by 5–80 Hz light stimulation at 470 nm (Fig. 4b). Selective light stimulation of these PV neurons in the NAc during the EPM task led to more avoidance behavior in PV-ChR2 mice compared with PV-mCherry controls, illustrated by a significantly lower number of entries and markedly less time spent in the anxiogenic open arms (Fig. 4c, d). The OFT led to similar results: light stimulation of PV neurons in PV-ChR2 mice led to less exploration time in the center of the open-field apparatus compared with the PV-mCherry control mice (Fig. 4e) without any difference in locomotion between the two groups during each five-min epoch (Fig. 4f).

In order to further determine the causal role of accumbal PV activity in avoidance behavior related to anxiety states, we expressed hM_4_D_i_ in PV neurons by bilateral injection of Cre-dependent AAV-DIO-hM_4_D_i_-mCherry into the NAc shell of PV-Cre mice. Mice were given a 10-min OFT followed by a 5-min EPM test (Fig. 4g). Co-localization of 76.1 % of PV cells with hM_4_D_i_ was verified by immunostaining (Fig. 4h). Representative OFT and EPM heat maps for both PV/mCherry (control) and PV/hM_4_D_i_ groups are shown in Fig. 4i–k: hM_4_D_i_ mice showed significantly greater center exploration, relative to mCherry controls. Consistent with the OFT findings, hM_4_D_i_ mice showed greater exploration of the open arms compared with the control mice, reflecting an inappropriate avoidance coping behavior (Fig. 4l–n). We confirmed that activation of NAc^PV^ neurons could suppress the firing of neighboring MSNs (Fig. S4a, b), so we then examined the impact of activation of D2 MSNs alone on anxiety-like behavior (Fig. S4c). We found that, compared with eYFP controls, optogenetic activation of D2 MSNs reduced entries to the central area in the open-field and to the open arms in the elevated-plus maze (EPM) (Fig. S4e, h), but did not influence total exploration time in either of these open areas (Fig. S4d, g). However, light stimulation of D2 MSNs in the NAc markedly decreased locomotor their activity (Fig. S4f), which is consistent with the prior studies [47, 48]. Taken together, these data suggest that activity of PV neurons within the NAc is required for execution of appropriate avoidance behavior to buffer the stress response evoked by anxiogenic environments.

### Inputs to NAc^PV^ neurons originate predominantly from the anterior dorsal BNST (adBNST)

**Fig. 5:**
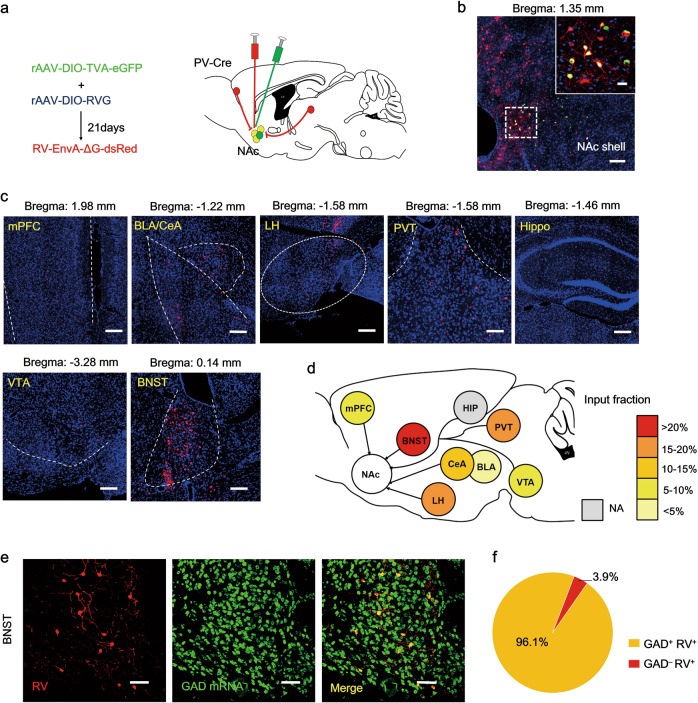
Monosynaptic GABAergic inputs to sNAc^PV^ neurons mainly stem from the adBNST. **a** Schematic showing sNAc injections of AAV-Ef1α-DIO-TVA-eGFP (AAV2/9) virus and AAV-Ef1α-DIO-RG (AAV2/9) on day 1 and RV-EvnA-DsRed on day 21 in PV-Cre mice to retrogradely trace input neurons (red) to NAc shell (yellow, starter neurons). **b** RV-mediated transsynaptic retrograde tracing of NAc inputs; fluorescence images of NAc region (coronal diagram) in PV-Cre mice (*n* = 3 mice); scale bar, 100 μm; *inset*, enlarged view of the region in the dotted white box, showing starter cells (yellow, expressing both eGFP and DsRed, indicated by white arrowheads; scale bar, 50 μm.) **c** Typical coronal-section planes with distance (anterior-posterior) from bregma showing dsRed-expressing presynaptic neurons retrogradely labeled by NAc injection of virus in PV-Cre mice; scale bar, 100 μm; HIP hippocampus, BLA basolateral amygdala, CeA central amygdala, PVT paraventricular thalamus, mPFC medial prefrontal cortex, LH lateral hypothalamus, VTA ventral tegmental area, BNST bed nucleus of the stria terminalis. **d** Overview of inputs to NAc PV neurons (*n* = 3 mice per region giving a total of 12–39 slices). **e** Sections were co-stained with antibodies against RFPn (RV) (red) and riboprobes (green) for *Gad1/2*; scale bar, 50 μm. **f** Approximately 96.1% of RV-infected neurons were co-labeled for *Gad1/2* in the adBNST (*n* = 12 slices, from 3 mice).

We next investigated whether the BNST was upstream of the NAc^PV^ neurons and if it is the region where anxiety-like avoidance coping behavior is integrated. Cre-dependent, rabies-virus-based whole-brain monosynaptic tracing was performed to analyze upstream regions that innervate NAc^PV^ neurons. We injected PV-Cre mice with Cre-dependent AAVs expressing the avian EnvA receptor (TVA) and rabies virus envelope glycoprotein (RG) in combination with theΔG-dsRed (EnvA) rabies virus (RV) (Fig. 5a). We found that a Cre-dependent helper virus combined with RV expressing dsRed labeled 84% of NAc^PV^ neurons (GFP^+^ cells, Fig. 5b). Our results showed that the dominant inputs to NAc^PV^ neurons were from the anterior dorsal part of the nucleus of the stria terminalis (adBNST) (52%, Fig. 5c, d), a classic GABAergic anxiety-associated region. Other brain regions that provided inputs to NAc^PV^ neurons included the basolateral amygdala (BLA, 4.34%), central amygdala (CeA, 10.27%), media prefrontal cortex (mPFC, 5.7%) as well as the ventral tegmental area (VTA, 6.07%). NAc^PV^ neurons also received monosynaptic inputs from reward-associated components, such as the paraventricular thalamus (PVT, 19.6%) and lateral hypothalamus (LH, 16.07%) [49]; no projections from the Hippocampus (HIP, 0%) were found (Fig. 5c, d). In situ hybridization experiments demonstrated the co-expression of Gad 1/2 and dsRed in aBNST (96.1%, Fig. 5e, f). These findings indicate that NAc^PV^ neurons were modulated under a GABAergic network. A negative control was performed with no injection of RG into the NAc shell, and no RV expressing dsRed labeled cells were found in the above brain regions (Fig. S5).

### Optogenetic activation of adBNST GABAergic inputs to the sNAc^PV^ neurons rescued stress-induced excessive avoidance behavior

**Fig. 6:**
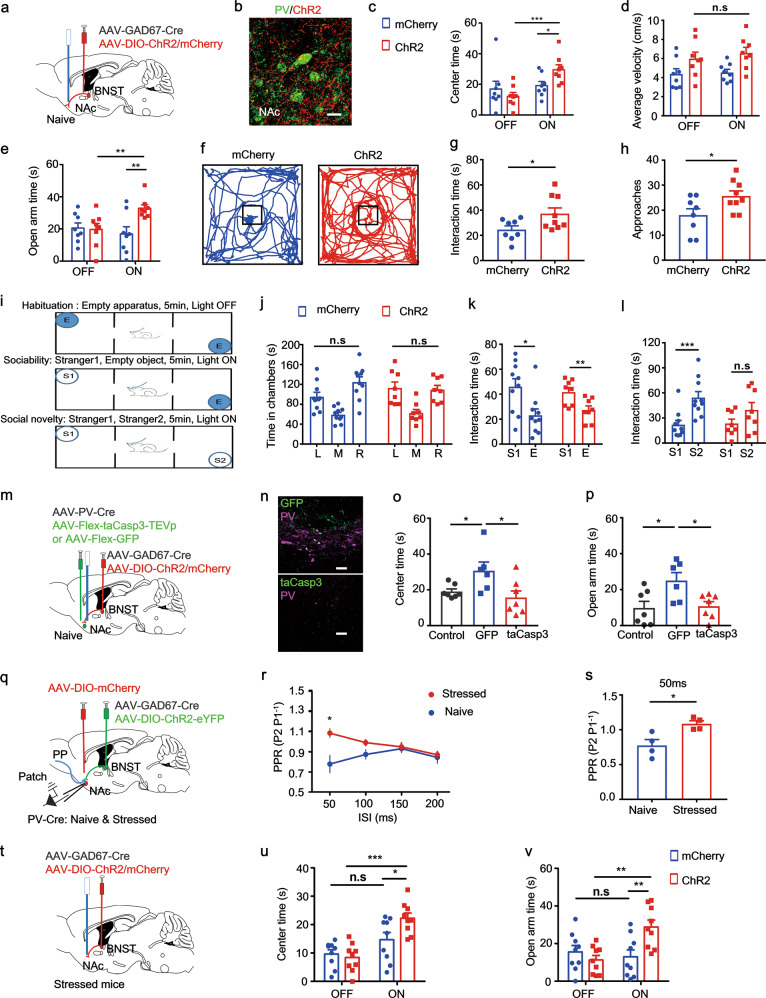
Optogenetic stimulation of adBNST GABAergic terminals in sNAc produces an anxiolytic effect, which is mediated by sNAc^PV^ neurons. **a** Schematic showing optogenetic manipulation of adBNST GABAergic afferent fibers that innervate NAc^PV^ neurons. **b** Representative image showing adBNST GABAergic afferent axons innervating the PV neurons within NAc shell. Scale bar, 10 μm. **c**–**d** Mean time spent in the center and average velocity in OFT (*n* = 8 mice per group, unpaired *t* -test, for **c**, ChR2: OFF vs. ON, *t* = 4.244, *P* = 0.0008; ON: mCherry vs. ChR2, *t* = 4.484, *P* = 0.0263; for **d**, *t* = 0.6661, *P* = 0.5162) with or without optogenetic stimulation of adBNST GABAergic afferent axons innervating PV neurons within NAc shell. **e** Mean time spent in the open arms in EPM (*n* = 8 mice per group, unpaired *t* test, ChR2: OFF vs. ON, *t* = 3.009, *P* = 0.0094; ON: mCherry vs. ChR2, *t* = 3.160, *P* = 0.0070). **f** Representative mouse tracks during the novelty object interaction for both mCherry and ChR2 groups. **g**–**h** Mean interaction time and approaches in mCherry and ChR2 groups (*n* = 8 per group, unpaired *t* test, for **g**, *t* = 2.286, *P* = 0.0372; for **h**, *t* = 2.343, *P* = 0.0333). (i) Schematic showing three-chamber social interaction tests. (j) Time in each chamber in habituation trials, L: Left; M: Middle; R: Right (n = 8 per group, paired *t* test, mCherry: *t* = 1.543, *P* = 0.1572; ChR2: *t* = 0.1754, *P* = 0.8657). **k** Interaction time with a stranger mouse (S1) or an empty cage (E) in sociability trials (*n* = 8 per group, paired *t* test, mCherry: *t* = 3.245, *P* = 0.0101; ChR2: *t* = 4.514, *P* = 0.0028). **l** Interaction time with the stranger mouse (S1) or the second stranger mouse (S2) in social novelty trials (*n* = 8 per group, paired *t* test, mCherry: *t* = 6.541, *P* = 0.0001; ChR2: *t* = 1.565, *P* = 0.1615). **m** Schematic showing viruses injected into the adBNST and NAc shell. Three groups: Control, BNST^mCherry^-NAc^GFP^; GFP, BNST^ChR2^-NAc^GFP^; taCasp3, BNST^ChR2^-NAc^taCasp3^. In each mouse, an optical fiber was implanted above the NAc shell for optogenetic illumination of adBNST GABAergic outputs. **n** Representative images showing selective deletion of PV neurons in the NAc shell; scale bar, 100 μm. **o**–**p** Mean time spent in the center (*n* = 6–7 mice per group, unpaired *t* test, GFP vs. Control: *t* = 2.410, *P* = 0.0346; taCasp3 vs. Control: *t* = 2.460, *P* = 0.0317) and in open arms (*n* = 6–7 mice per group, unpaired *t* test, GFP vs. Control: *t* = 2.660, *P* = 0.0222; taCasp3 vs. Control: *t* = 2.880, *P* = 0.0150). **q** Schematic showing PPR of eIPSPs recorded from PV neurons within the NAc shell in wild-type and stressed PV-Cre mice during optogenetic stimulation of adBNST GABAergic afferents around PV neurons. **r** PPR of eIPSPs plotted as a function of interspike intervals (ISIs); **s** mean PPR measured at 50 ms interspike interval. (*n* = 4 from 3 mice per group, unpaired *t* test, *t* = 3.247, *P* = 0.0175). **t** Schematic showing optogenetic stimulation of adBNST GABAergic afferents around PV neurons in the sNAc on stressed mice. **u**–**v** Mean time in the center and open arms (*n* = 9 mice per group, unpaired *t* test, for **u**, ChR2: OFF vs. ON, *t* = 5.540, *P* < 0.0001; ON: mCherry vs. ChR2, *t* = 2.396, *P* = 0.0292; for **v**, ChR2: OFF vs. ON, *t* = 3.961, *P* = 0.0011; ON: mCherry vs. ChR2, *t* = 3.047, *P* = 0.0077).

Next, we investigated the impact of the connection between adBNST GABAergic neurons and sNAc^PV^ cells on producing anxiety-like behavior. We virally expressed GAD67-Cre and Cre-dependent channelrhodopsin-2 (ChR2) in adBNST GABAergic neurons and visualized sNAc^PV^ neurons by injection of adeno-associated viruses (AAVs) encoding the fluorophore mCherry into PV-Cre mice (Fig. S6a). Co-staining results revealed that the majority of neurons expressing GAD^+^ also co-expressed ChR2 (Fig. S6b, c). We recorded evoked IPSPs from PV neurons within the NAc shell by illumination of adBNST afferent axon fibers, which was completely blocked by 20 μM bicuculline, implying a GABAergic monosynaptic input to the sNAc^PV^ neurons (Fig. S6d, e). The mean latency was 4.07 ± 0.7 ms, in line with monosynaptic transmission (Fig. S6f, right). These data suggest a direct functional GABAergic input to the sNAc^PV^ neurons. We further targeted the function of this GABAergic input to sNAc^PV^ neurons by virally expressing Cre-dependent ChR2 and GAD67-Cre in adBNST neurons followed by light stimulation of the terminals within sNAc (Fig. 6a, b). Blue light stimulation (ON) resulted in less avoidance of both OFT central area (Fig. 6c) and EPM open arms (Fig. 6e) in the ChR2-expressing naive mice (ChR2), compared with mCherry-carrying controls (mCherry), with no difference in locomotor activity between groups (Fig. 6d). Approaching a novel environment or object is considered anxiogenic/risky [50], and we therefore investigated the role of this GABAergic circuit when approaching a novel object (Fig. 6f). Light stimulation of the terminals within sNAc led to more interaction with, and also a higher frequency of approaches to, the novel object (a Rubik’s cube; Fig. 6g–h). Furthermore, we used a three-chamber social test to determine whether sociability was influenced by light stimulation of these GABAergic inputs to the sNAc^PV^ neurons (Fig. 6i). Mice showed no preference for the left or right chamber in the habituation trial (Fig. 6j). Light stimulation of the terminals within the sNAc during the sociability trials resulted in mice spending more time interacting with the stranger mouse than time in the empty chamber, which suggested that activation of this circuit had no significant effect on sociability (Fig. 6k). However, during social novelty trials, light stimulation of the terminals within the sNAc resulted in the natural preference for the novel stranger (S2) [51, 52] over the familiar one (S1) being inhibited (Fig. 6l). This lack of preference for a novel partner (S2) over the familiar one (S1) suggests that activation of all the GABAergic inputs had some effect on social novelty recognition; however, basal social preference did not change when without light stimulation of the GABAergic terminals (Fig. 6l). In summary, activation of the adBNST GABAergic inputs to sNAc^PV^ neurons reduced avoidance of risky/anxiogenic stimuli and had some influence on social behavior.

We then tested whether, under similar conditions, we would obtain similar results without adBNST neurons innervating sNAc^PV^. AAV2/9-FLEX-taCasp3-TEVp and PV-Cre viruses were injected into the sNAc to selectively kill PV^+^ neurons; Cre-dependent ChR2 and GAD67-Cre were both injected into the adBNST of mice to specifically activate this new BNST-NAc GABAergic circuit (Fig. 6m); immunostaining confirmed that most of the PV^+^ neurons were killed by taCasp3 compared with the control virus (Fig. 6n). Terminals in the NAc shell were stimulated once again and we found that reduced avoidance of EPM open arms and OFT center was now effectively blocked during the light ON phase (Fig. 6o, p). These findings suggest a significant role of adBNST GABAergic projections to the NAc^PV^ neurons in anxiety-like behavior. To examine the presynaptic effect of GABA release on the NAc^PV^ neurons, we recorded two consecutive eIPSPs, which were separated by varying interspike intervals to calculate the paired-pulse ratio (PPR), during light stimulation of the adBNST GABAergic afferents to sNAc (Fig. 6q). We found that the PPR was significantly increased in the stressed mice compared with their naive littermates at 50 ms interspike interval (Fig. 6r, s). The increased PPR in the stressed mice suggests an impaired presynaptic GABA release at adBNST to sNAc synapses, further indicating these sNAc^PV^ neurons are disinhibited by GABAergic inputs from the adBNST under a chronic stress state.

Based on these findings, we then tested whether activation of the adBNST GABAergic afferents to sNAc^PV^ neurons could rescue the pathological anxiety-like behavior in the stressed mice. We injected AAV-GAD67-Cre and either DIO-ChR2 or DIO-mCherry into the adBNST of stressed mice and implanted an optical fiber above the sNAc (Fig. 6t). Light stimulation of adBNST GABAergic afferents in sNAc significantly increased the time spent in the OFT center and open arms of the EPM (Fig. 6u, v), which implies that excessive avoidance behavior related to the anxious state was rescued. Taken together, these findings indicate that in anxious states, the adBNST may send a disinhibition input to the sNAc^PV^ neurons, leading to excessive avoidance of the threatening locations.

### sNAc^PV^ neurons that control avoidance of anxiogenic stimuli are innervated predominantly by somatostatin afferents from adBNST

**Fig. 7:**
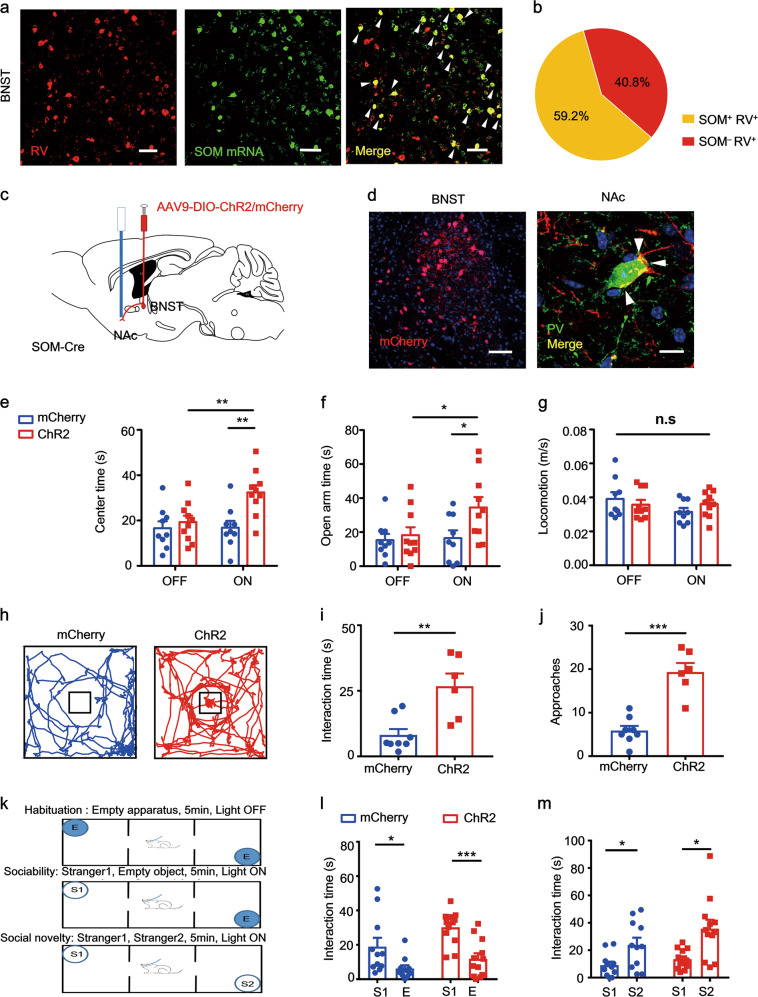
adBNST^SOM^ inputs to sNAc^PV^ neurons regulate avoidance of anxiogenic stimuli. **a** Sections co-stained with antibodies against RFPn (RV) (red) and riboprobes (green) for SOM; scale bar, 50 μm. **b** Approximately 60% of RV-infected neurons co-expressed SOM in the adBNST (*n* = 3 mice). **c** Schematic showing viral and optogenetic strategy for targeting specific somatostatin projections from adBNST to the sNAcPV neurons. **d** Representative images showing mCherry expression in adBNST (*n* = 3 mice, scale bar, 50 μm) and detection of the SOM afferent fibers around PV neurons within NAc shell (*n* = 3 mice, scale bar, 10 μm). **e**–**f** Mean time spent in the center (*n* = 9–10 per group, unpaired *t* test, ChR2: OFF vs. ON, *t* = 3.105, *P* = 0.0061; ON: mCherry vs. ChR2, *t* = 3.531, *P* = 0.0026) and open arms (*n* = 9–10 per group, unpaired *t* test, ChR2: OFF vs. ON, *t* = 2.105, *P* = 0.0496; ON: mCherry vs. ChR2, *t* = 2.302, *P* = 0.0342). **g** Mean velocity with or without light stimulation of the SOM terminals in sNAc (*n* = 9–10 per group, unpaired *t* test, ChR2: OFF vs. ON, *t* = 0.1105, *P* = 0.9132; ON: mCherry vs. ChR2, *t* = 1.407, *P* = 0.1773). **h** Representative mouse tracks during the novelty object interaction for both mCherry and ChR2 mice. **i**–**j** Mean interaction time and approaches in mCherry and ChR2 groups (*n* = 8–6 per group, unpaired *t* test, for **i**, *t* = 3.772, *P* = 0.0027; for **j**, *t* = 6.194, *P* < 0.0001). **k** Schematic showing social interaction tests. **l**–**m** Interaction time in both mCherry control and ChR2 mice (*n* = 11–12 per group, paired *t* test, for **l**, mCherry: *t* = 2.457, *P* = 0.0338; ChR2: *t* = 4.463, *P* = 0.0010; for **m**, mCherry: *t* = 2.245, *P* = 0.0486; ChR2: *t* = 2.992, *P* = 0.0122).

We further investigated the subtype of the adBNST GABAergic inputs to the sNAc^PV^ and identified that a majority of adBNST neurons sent dense somatostatin (SOM) afferents (59.2%) to innervate PV neurons within the NAc shell (Fig. 7a, b) and co-expressed with prodynorphin (PDYN) (Fig. S7a). In addition, 37.1% of the GABAergic afferents were PDYN positive but SOM negative (Fig. S7a). Furthermore, we found that 3*.*9% of the GABAergic afferents were neither SOM nor PDYN positive (Fig. S7a). Meanwhile, we found that, amongst these adBNST GABAergic projection neurons, 16.5% of the cells were corticotropin-releasing hormone (CRH) and SOM double positive, 42.1% were SOM positive but CRH negative and 12.6% were CRH positive but SOM negative (Fig. S7b). Furthermore, we found that neither PV, choline acetyltransferase (ChAT) nor vesicular glutamate transporter (VGluT) was co-expressed with these adBNST GABAergic inputs to the sNAc^PV^ (Fig. S7c–e). In summary, these findings suggest that these adBNST SOM afferents were also co-expressing either PYDN, CRH or both.

Next, we investigated the function of these SOM projections in regulating avoidance of the anxiogenic stimuli. We virally expressed Cre-dependent channelrhodopsin-2 (ChR2) in adBNST SOM neurons with SOM-cre mice (Fig. 7c) and the afferent fiber signals were detected around PV neurons in the NAc shell (Fig. 7d, white arrowheads). Optogenetic stimulation of these fibers in the sNAc significantly reduced avoidance of both OFT central area (Fig. 7e) and EPM open arms (Fig. 7f) but did not affect locomotion (Fig. 7g). We then investigated the role of this specific neuronal circuit when approaching a novel object (Fig. 7h). Light stimulation of SOM afferents to the sNAc^PV^ neurons led to more interaction with, and also a higher frequency of approaches to, the novel object (a Rubik’s cube; Fig. 7I, j). Furthermore, we used a three-chamber social test to see whether sociability was influenced by light stimulation of the SOM fibers (Fig. 7k). Light stimulation of adBNST^SOM^ projections to sNAc^PV^ neurons had no effect on sociability and social novelty preference as mice interacted more with a stranger (S1) than the empty chamber, and they spent more time with a novel stranger (S2) compared with the first stranger (S1) (Fig. 7l, m). These data suggest that activation of the adBNST^SOM^ inputs to sNAc^PV^ neurons reduced avoidance of risky/anxiogenic stimuli but did not influence social behavior.

## Discussion

Chronic stress leads to long-term changes in brain structure and function, which increases the incidence of stress-related disorders, such as anxiety [4]. Anxiety disorders are the most prevalent mental disorders and are associated with immense social health care costs [22]. A central symptom is avoidance behavior, which also acts as a reinforcer of the anxious state [24, 53]. It is vital then to understand the underlying cellular and circuitry mechanisms underpinning this type of avoidance behavior, which could result in new methods to break the cycle of anxiety-avoidance.

A critical role in stress response and anxiety has been attributed to the BNST in both rodent and human studies [3, 54, 55]. The NAc is a key component of the brain “reward” circuits [56–58] in emotional and motivated actions and its dysfunction has been strongly implicated in emotional disorders [59, 60], especially in exerting a dominant influence on anxiety [10, 61, 62]. Although one study noted a projection from BNST to NAc [11], knowledge of the function of the link between the stress response neurocircuitry and reward circuitry not known.

Our resting fMRI findings indicate that, besides the high correlation between the BNST and amygdala (Fig. S1a), increased connectivity was observed between BNST and NAc under a chronic-stress-induced anxious state (Fig. 1e–g). LFP coherences in *theta* and *gamma* bands between these two regions were also significantly increased (Fig. 1j), confirming an intrinsic link between the stress response region and the reward circuit component under anxious state. Our finding is consistent with studies by Joshua Gordon’s group looking at other brain regions, which suggest that under anxiety states, both *theta* and *gamma* oscillations were increased [63, 64]. Much evidence now implicates both BNST and NAc involvement in anxiety and addiction, two highly prevalent stress-related disorders [4, 65], implying interconnectivity between the two areas. As research uncovers more details of the functional connectivity regarding specific neuronal subpopulation projections within BNST-NAc circuitry, we may better understand the mechanisms underlying these two psychiatric diseases and the BNST to NAc circuit may become relevant for drug targets for therapeutic interventions. Our fMRI results also showed a significant decrease in the PAG-HIP circuit in chronically stressed mice (Fig. S1a, middle), which suggests a dissociation between these two areas during anxiety states. In summary, we can envision that the increased synchronization of BOLD signals in BNST and NAc may potentially be used as an imaging marker for the diagnosis of anxiety disorders in the future.

LFP recordings showed a marked decrease in local *theta* power, both in the NAc and BNST of stressed mice (Fig. 1k, l), indicating PV cell involvement within these two regions as previous studies found a correlation between PV activity and local *theta* changes [37, 38]. However, only the NAc shell contained PV cell bodies (Fig. 2a) and the patch-clamp data found that these accumbal PV cells were hyper-excitable under an anxious state (Fig. 2d, e), confirming the surprising role of accumbal PV interneurons in behavior related to anxiety, given their low occurrence in the region. Anxious states are reflected by the reverse correlation between neuronal activity and open-arm exploration time [66]. We therefore performed in vivo calcium signal recordings, and further confirmed that activation of accumbal PV neurons is negatively correlated to the exploration time of anxiogenic EPM open arms (Fig. 3n).

Indeed, other observations in our present study also support the importance of sNAc^PV^ neurons in control of anxiety-like behavior. First, we used *in vivo* single-unit recordings to confirm the relationship between anxiogenic stimuli and the activity of accumbal PV neurons. Amongst our recorded neurons, a type of fast-spiking (FS) unit showed preferential activity in an anxiety-inducing environment (open arms, Figs. 3c, d, S2a). Most fast-spiking neurons within the NAc have previously been identified as PV neurons, which strongly suggests that accumbal PV neurons were engaged in this anxiogenic-related behavior. Second, in vivo genetic manipulation of PV-neuronal activity in both healthy and chronic-stress models further highlighted the importance of these neurons in encoding anxiety-like behavior (Figs. 2i–k and 4). Taken together, these findings greatly extend previous observations of NAc involvement in anxiety-like information processing. This is the first study to reveal real-time PV activity in the accumbens during encoding of anxiety-like behavior in free exploration of aversive spaces without prior training, and indicates that activity of sNAc ^PV^ neurons was required for anxiety-like avoidance.

Cholinergic interneurons (CINs) are another well-characterized class of interneuron within striatal areas [67]. CINs display a range of firing frequencies (0–3.06 Hz) with mean and median firing rates of 0.74, respectively [68, 69] and are thus non-fast spiking neurons. Due to the relationship between the fast-spiking neuronal activation and anxiogenic stimuli (Fig. 3), we suggest that accumbal CINs are not majorly involved in anxiety-like behavior.

Previous studies have shown that the NAc receives intermingled glutamatergic and dopaminergic inputs from a variety of forebrain regions, including the amygdala [23], hippocampus [21], thalamus [22, 44], ventral tegmental area [19] and the prefrontal cortex [45]. Using Cre-dependent, rabies-virus-based whole-brain monosynaptic tracing strategy and electrophysiological recordings from brain slices, we demonstrated that NAc PV cells were specifically innervated by the GABAergic afferents stemming from the adBNST (Figs. 5 and S6d). To our best knowledge, this is the first study to map novel neural circuitry specifically innervating sNAc^PV^ neurons. Although a recent study found that light-evoked activation of ventral hippocampus inputs to NAc resulted in sNAc^PV^ cell regulation of cocaine-seeking behavior, a cell-specific monosynaptic tracing strategy was not used in their study to show the anatomic connection [21]. Therefore, we speculate that the PV response to ventral hippocampus afferent activation may be indirect. On the other hand, it is believed that the predominant neuron types in the BNST are GABAergic ones [40], but the PV neurons within the NAc only account for ~4% of total neuron populations [21]. Therefore, the specific projections from BNST to sNAc^PV^ appear to be relatively few (Fig. 5e).

A previous study has shown that anterior BNST-associated activity exerts anxiolytic influence on anxious states [28]. Several of our current findings are consistent with this conclusion: (1) specific activation of these afferents from the adBNST resulted in a robust inhibition of accumbal PV activity (Fig. S6d) and reduced avoidance coping behavior in response to anxiogenic stimuli (Fig. 6c, e) without any differences in locomotor activity between the ChR2-manipulated group and mCherry control group (Fig. 6d); 2) when PV function was ablated, the previously observed reduced avoidance behavior was also abolished (Fig. 6m–p); 3), whereas elevating the activity of GABAergic inputs to the sNAc^PV^ neurons rescued anxiety-like excessive avoidance behavior, representing an anxiolytic effect (Fig. 6t–v). Changes of paired-pulse ratio (PPR) have been widely accepted to reflect presynaptic neurotransmitter release, including glutamate and GABA [70]. In our chronic stress model, we found there was an increase in the PPR in the adBNST-- sNAc circuit, indicating impaired release of GABA onto sNAc^PV^ cells following light activation of the GABAergic terminals in the sNAc (Fig. 6q–s). Combining these findings, we summarize that adBNST sends GABAergic inputs to sNAc to control avoidance behavior, which is mediated by sNAc^PV^ neurons.

To some surprise, we found the fact that chronic stressors could both increase functional connectivity between BNST and NAc (Fig. 1g) and attenuate the inhibitory synaptic transmission at adBNST^GABA^ to sNAc^PV^ synapses (Fig. 2d). We suggest that the increase in fMRI connectivity reflects the overall changes between BNST and NAc and that this masks the attenuation occurring at specific synapses. Previous studies have also demonstrated that neuronal activities and BOLD signals can be reversed [71–73].

We further identified that most of these GABAergic afferent axon fibers that innervate sNAc^PV^ cells were somatostatin positive (Fig. 7a, b). Activation of these SOM inputs produced similar anxiolytic effects on both OFT and EPM measures (Fig. 7e, f) without change in locomotion (Fig. 7g); the novel object approaching test further demonstrated anxiolytic effects of activation of these SOM inputs from the adBNST (Fig. 7h–j). Based on the above, we conclude that we identified a new somatostatin positive pathway that engages the adBNST via a population of accumbal PV fast-spiking neurons for anxiety-like avoidance coping behavior. However, further study is needed to investigate whether the BNST also projects to NAc D2 cells and whether this projection participates in anxiety-like behaviors.

PV activity has been implicated in contributing to the *theta* rhythms in the mPFC and the hippocampus [37, 38] and our results also show a marked decrease in *theta* rhythm on stressed mice both in the NAc and the BNST (Fig. 1k, l). In addition, electrophysiological recordings from freely moving mice confirmed consistently lower local *theta* power in healthy animals during exploring the anxiogenic open arms (unrelated to locomotion, Fig. S2c, d), suggesting a strong correlation between *theta* power changes and anxious states. We reason that accumbal PV neurons exhibit high excitability under anxious states and, therefore, highly activated PV cells contribute to *theta* oscillations changes either in BNST or NAc. Further study is needed to determine whether PV neuronal activation in the accumbens is the main driver for the decrease in *theta* oscillation within these two structures (Fig. 1k, l), although in the present study the increase in the coherence between FS spikes and LFP in *theta* range does suggest that this may be the case (Fig. S2f).

In conclusion, our results provide strong evidence for accumbal PV neurons driving anxiety-like avoidance coping behavior and may provide a new basis for the therapeutic purpose of pathological anxiety. Despite being a relative minor (~4%) component of all NAc neurons [21], this population has a robust anxiety-like behavioral effect. These accumbal PV cells are innervated by a long GABAergic-projecting SOM input stemming from the adBNST. Anxiety represents a brain state: our study uncovered a new circuit mechanism, precisely defined by the neuronal types involved, by which the stress response brain region orchestrates the reward circuit component to exert direct effects on anxious states. Our findings may help to explain why anxiety and addiction are highly comorbid, although these two common psychiatric disorders engage emotion and reward circuits, respectively.

## Supplementary information

SUPPLEMENTAL INFORMATION

Figure Sup 1

Figure Sup 2

Figure Sup 3

Figure Sup 4

Figure Sup 5

Figure Sup 6

Figure Sup 7
