# Supplementary material for: A new GABAergic somatostatin projection from the BNST onto accumbal parvalbumin neurons controls anxiety
Source: Mol Psychiatry. 2020 Jun 18;26(9):4719–41. doi: 10.1038/s41380-020-0816-3 (PMC8589681; doi:10.1038/s41380-020-0816-3)
Supplement: Supplementary file 1 — SUPPLEMENTAL INFORMATION [file 41380_2020_816_MOESM1_ESM.docx]

**SUPPLEMENTAL INFORMATION**

Figure Sup 1. Resting-state fMRI functional connectivity analysis.

Figure Sup 2. Accumbal *theta* power is coherent with fast spiking neuronal activitiy.

Figure Sup 3. NAc Ca^2+^ transients of different eYFP-tagged neuron populations in the EPM.

Figure Sup 4. *In vivo* recording of optogenetically-tagged NAc^PV^ and MSN cells.

Figure Sup 5. Negative control for retrograde tracing of the input neurons that send afferents to sNAc^PV^ neurons.

Figure Sup 6. adBNST GABAergic sent direct monosynaptic input to the NAc^PV^ neurons.

Figure Sup 7. Fluorescence *in situ* hybridization or immunohistochemistry staining for the identities of the BNST neuronal types innervating downstream sNAc^PV^ neurons.

**Sup 1.** **Resting-state fMRI functional connectivity analysis**

(a) BOLD synchronization in Amy-BNST, PAG-HIP and PFC-NAc in naive and stressed littermates (n = 5-7 mice per group, unpaired *t* test, left, *t* = 3.143, *P* = 0.0035; middle, *t* = 2.316, *P* = 0.0267; right, *t* = 1.474, *P* = 0.1502). (b) Heat maps showing a correlation of resting-state fMRI BOLD signal across brain regions viewed in coronal sections with a seed (ROI) in NAc.

**Sup 2. Accumbal *theta* power is coherent with fast spiking neuronal activitiy**

(a) Left, horizontal and vertical arms represent closed and open arms, respectively; right, Z-scores of FS neurons during movement from the open to closed arms (nonparametric Kolmogorov-Smirnov test, n = 5, *P* = 0.047). (b) There was no difference in individual Non-FS neuron firing rates from open to closed arms. (c) LFP within NAc shell in open and closed arms; inset, difference in the local theta activity between open and closed arms (Paired *t* test, n = 21, *t* = 2.083, *P* = 0.0251). (d) Correlation between locomotion and FS neuron LFP. (e) Average peri-event LFP spectrogram showing changes in the *theta* band during movement from the closed to open arms or from the open to closed arms. (f) Spike-LFP coherence at *theta* power of FS neurons and Non-FS neurons (Mann-Whitney rank sum test, n of FS = 5, n of Non-FS = 28, *t* = 141, *P* = 0.007).

**Sup 3. NAc Ca^2+^ transients of different eYFP-tagged neuron populations in the EPM**

(a-c) Sections were co-stained with antibodies against eGFP (green) and riboprobes (red) for *D1R mRNA* (A, *left*)*, D2R mRNA* (b, *left top*) *and PV mRNA* (c, *left*); scale bar, 100 μm; enlarged view of the white box region showing cells co-expressing *D1R mRNA* (a, *middle)*, *D2R mRNA* (b, *left bottom)*, *PV mRNA* (c, *middle*) and GCaMP6m, respectively; scale bar, 10 μm, n = 9 slices from 3 mice per group; *right*, pie chart showing the respective co-expressed quantities of each mRNA type (*D1R mRNA* 91.6%*, D2R mRNA* 96.1%*, and PV mRNA* 84%). (d-f) *Top,* heatmaps of normalized NAc Ca^2+^ activity from different eYFP-tagged neuron populations in the EPM, binned by time (s) from the EPM crossing point (*inset*, red point). *Inset,* horizontal and vertical arms represent open and closed arms, respectively; *bottom*, normalized NAc Ca^2+^ transients of different eYFP-tagged neuron populations in the EPM open arms compared to closed arms (Wilcoxon test, n = 250).

**Sup 4. *In vivo* recording of optogenetically-tagged NAc PV and MSN cells**

Examples of (a) an optogenetically-tagged NAc PV^+^ FS and (b) MSN recorded *in vivo*. From left to right, example spikes (gray traces) with superimposed mean waveforms (red/black traces) *(left*) in Light ON conditions, raster plots showing normalized short-latency light-driven spiking of FS units and decreased spiking of the MSN (*middle*), and their spike firing probabilities (*right*). The sharp peak at short latency identifies this FS unit as an optogenetically-tagged PV^+^ cell. (c) Schematic showing optogenetic manipulation of NAc^D2^ neurons. (d-f) Mean time spent in the center, entries to the center, and average velocity in the OFT (n = 7-8 mice per group, unpaired *t* test, for d, ChR2: OFF vs. ON, *t* = 2.055, *P* = 0.0590; for e, *t* = 2.503, *P* = 0.0253; for f, ChR2: OFF vs. ON, *t* = 5.159, *P* = 0.0001). (g-h) Mean time spent in the open arms and entries to the open arm in the EPM (n = 7-8 mice per group, unpaired *t* test, for h, ChR2: OFF vs. ON, *t* = 2.679, *P* = 0.0180).

**Sup 5. Negative control for retrograde tracing of the input neurons that send afferents to sNAc^PV^ neurons**

(a) Schematic showing injection of AAV-Ef1α-DIO-TVA-eGFP (AAV2/9) virus on day 1 and RV-EvnA-DsRed on day 21 into the sNAc of PV-Cre mice to retrogradely trace the input neurons (red) to NAc shell (yellow, starter neurons). (b) Fluorescence images of NAc region (coronal diagram) in PV-Cre mice (n = 4 mice), scale bar, 100 μm; *inset*, enlarged view of the white box region showing starter cells (yellow, expressing both eGFP and DsRed. Scale bar, 50 μm.) (c) No DsRed signals found in related brain regions.

**Sup 6. adBNST GABAergic sent direct monosynaptic input to the NAc^PV^ neurons**

(a) Schematic showing the strategy for selective optogenetic manipulation of GABAergic afferents from aBNST onto sNAc^PV^ neurons and recording postsynaptic inhibitory potentials (IPSP). (b-c) Representative image showing that most ChR2 positive cells in adBNST co-expressed GAD1/2 mRNA (n = 3 mice); scale bar, 100 μm. (d) *Top*, mean eIPSPs obtained from PV neurons within the NAc shell *(bottom*) was blocked by 20 μM bicuculline. (e) The eIPSP amplitude of PV neurons following different light stimulation duration from 1-20 ms. (f) Quantification of eIPSP amplitude (*left*, n = 7 cells from 4 mice) and latency (*right*, n = 7 cells from 4 mice).

**Sup 7. Fluorescence *in situ* hybridization** **or immunohistochemistry staining for the identities of the BNST neuronal types innervating downstream sNAc^PV^ neurons**

(a) *Left*, representative images showing co-expression of SOM, PDYN and RV signals in the aBNST, right, pie chart shows the neuronal percentage. (b) *Left*, representative images showing co-expression of SOM, CRH and RV signals in the aBNST, right, pie chart shows the neuronal percentage. (c-e) *Left*, representative images showing no expression of PV, ChAT or VGLUT in the aBNST; *right,* pie chart showing the RV positive cells were neither expressing PV, ChAT nor VGLUT. (n = 3 mice, scar bar, 50 um).
